# Epigenetic age prediction in semen – marker selection and model development

**DOI:** 10.18632/aging.203399

**Published:** 2021-08-10

**Authors:** Aleksandra Pisarek, Ewelina Pośpiech, Antonia Heidegger, Catarina Xavier, Anna Papież, Danuta Piniewska-Róg, Vivian Kalamara, Ramya Potabattula, Michał Bochenek, Marta Sikora-Polaczek, Aneta Macur, Anna Woźniak, Jarosław Janeczko, Christopher Phillips, Thomas Haaf, Joanna Polańska, Walther Parson, Manfred Kayser, Wojciech Branicki

**Affiliations:** 1 Malopolska Centre of Biotechnology, Jagiellonian University, Krakow, Poland; 2 Institute of Legal Medicine, Medical University of Innsbruck, Innsbruck, Austria; 3 Department of Data Science and Engineering, The Silesian University of Technology, Gliwice, Poland; 4 Department of Legal Medicine, Medical College, Krakow, Poland; 5 Department of Genetic Identification, Erasmus MC University Medical Center Rotterdam, Rotterdam, The Netherlands; 6 Institute of Human Genetics, Julius Maximilians University, Würzburg, Germany; 7 The Fertility Partnership Macierzynstwo, Krakow, Poland; 8 PARENS Fertility Centre, Krakow, Poland; 9 Central Forensic Laboratory of the Police, Warsaw, Poland; 10 Department of Legal Medicine, Santiago de Compostela, Spain; 11 Forensic Science Program, The Pennsylvania State University, University Park, PA 16802, USA

**Keywords:** semen, epigenetic age, DNA methylation, amplicon bisulfite sequencing, epigenetic age estimation

## Abstract

DNA methylation analysis is becoming increasingly useful in biomedical research and forensic practice. The discovery of differentially methylated sites (DMSs) that continuously change over an individual’s lifetime has led to breakthroughs in molecular age estimation. Although semen samples are often used in forensic DNA analysis, previous epigenetic age prediction studies mainly focused on somatic cell types. Here, Infinium MethylationEPIC BeadChip arrays were applied to semen-derived DNA samples, which identified numerous novel DMSs moderately correlated with age. Validation of the ten most age-correlated novel DMSs and three previously known sites in an independent set of semen-derived DNA samples using targeted bisulfite massively parallel sequencing, confirmed age-correlation for nine new and three previously known markers. Prediction modelling revealed the best model for semen, based on 6 CpGs from newly identified genes *SH2B2*, *EXOC3*, *IFITM2*, and *GALR2* as well as the previously known *FOLH1B* gene, which predict age with a mean absolute error of 5.1 years in an independent test set. Further increases in the accuracy of age prediction from semen DNA will require technological progress to allow sensitive, simultaneous analysis of a much larger number of age correlated DMSs from the compromised DNA typical of forensic semen stains.

## INTRODUCTION

Modification of DNA methylation (DNAm) is an important mechanism of epigenetic gene regulation. Dysregulation of DNAm has been observed in numerous diseases and consequently testing methylation patterns may have clinical value [1–3]. Moreover, the usefulness of DNAm analysis has also been recognized in forensic (epi)genetics, and practical forensic applications include identification of body fluids [4, 5], authentication of DNA samples [6, 7], differentiation of monozygotic twins [8, 9], prediction of lifestyle habits such as smoking and other forensically relevant extrinsic factors [10, 11], and in particular, the use of DNAm patterns to predict a person’s age [12–14].

The analysis of DNAm in forensic epigenetics can complement currently available methods of human identification and increase the use of DNA mainly for investigative intelligence purposes. DNA intelligence is applied to criminal cases without known suspects and allows the use of DNA to help find unknown suspects that cannot be identified with forensic STR profiling because their profiles are not already known to the investigators. The predictive power of information contained in the DNA is increasingly used in forensics, which has led to the establishment of the subfield of forensic DNA phenotyping [15]. Intelligence data obtained from DNA provides not only sex determination, but also inference of bio-geographic ancestry [16], prediction of some appearance traits - most notably pigmentation traits [17], and more recently, prediction of age [18]. All this information can be used to characterize an unknown person and provide investigative leads to focus and guide police investigation in their search for unidentified perpetrators [19].

Amongst DNA-based intelligence tools, predicting a person’s age from DNA can be particularly useful. Age information not only directly helps to trace unknown suspects, but can be an important factor in interpreting the results of appearance trait prediction, as several appearance traits such as hair loss in men are age-dependent. The discovery of differentially methylated sites (DMSs) that continuously change throughout an individual’s lifetime has led to the development of accurate methods of age prediction [12–14]. In terms of accuracy, these approaches far exceed DNA methods previously developed for predicting age in forensics, based on telomere length or sjTREC analysis [20, 21]. In addition, it has been shown that DNAm patterns are highly stable and thus allow age prediction in forensic material typically subjected to degradation [22–24]. However, the wider use of epigenetic age estimation in the forensic field is hampered by DNAm differences between cell types, and different DNAm marker sets, models and tools have become necessary to predict age in various forensically relevant tissues and body fluids [25–27]. Semen samples are frequently used for genetic testing in forensic DNA laboratories, particularly in sexual assault cases. However, previous epigenetic age prediction studies mainly focused on somatic cell types, while reports of age predictors for semen are few in number to date [28, 29]. Even in a large set of 353 carefully selected CpG markers, the groundbreaking work of Horvath showed the accuracy of predicting epigenetic age between different somatic tissues, with a median absolute difference error ranging between 1.5 years for the occipital cortex and 18 years for muscle. In sperm cells, no significant correlation was found, and the epigenetic clock predicted age at a significantly lower value than the true chronological age of the sperm donors [12].

Some studies indicate sperm cells have a very different pattern of age-related DNAm compared to somatic cells [30, 31]. In sperm cells, not only does DNAm evidently decrease with age in most genes, but telomere length does not decrease, in contrast to patterns observed in somatic cells [32]. In a recent study, Infinium HumanMethylation450 BeadChip array data were used to investigate semen samples collected from 329 donors. The final linear regression model included averaged DNAm levels across CpGs from 51 age-related regions and showed prediction accuracy in the test data set of MAE = 2.37 years [29]. Quantitative measurement of DNAm levels is technically challenging and DNA samples usually analysed in forensic laboratories are of low quality and quantity, which is further compromised by bisulfite conversion. Therefore, choosing the right technology for forensic applications is crucial. In particular, the use of microarray technology cannot efficiently analyze biological traces due to the low quality and small amount of DNA they yield.

The development of practical forensic methylation analysis tests that ensure high sensitivity is further hindered by the limited multiplexing capacity of current targeted DNAm detection technologies. Hence, analyzing the numerous CpGs from 51 regions suggested by Jenkins et al. [29] is currently impossible from crime scene DNA due to the lack of suitable technologies. In the search for efficient age DNA predictors from semen for forensic applications, aiming to reduce the number of DNA predictors to a minimal, Lee et al. (2015) conducted a marker discovery analysis in DNA from 12 sperm donors from 20 to 59 years of age. Lee used the Infinium HumanMethylation450 BeadChip array and discovered 24 potential epigenetic age predictors. The study proposed a predictive system based on analysis of three markers using a SNaPshot single base extension (SBE) protocol and a linear regression prediction model. These best semen age predictors were cg06304190 in the *TTC7B* gene, cg06979108 in *FOLH1B* (named *NOX4* on the Infinium HumanMethylation450 BeadChip array), and cg12837463 in *LOC401324* (no gene on the HumanMethylation450 array). The 3-CpG model predicted age with an error of ~5 years [28]. The prediction accuracy obtained with this model and tool in a follow-up forensic validation study was similar, with an MAE of 4.8 years [33].

In the current study, we first performed discovery of suitable semen age predictors via epigenome-wide screening for age-correlated DMSs using the Infinium MethylationEPIC BeadChip arrays (targeting over 850,000 CpGs), in bisulfite converted DNA from 40 semen samples collected from healthy men at age 24–58 years. The ten most promising candidate loci were validated together with the 3 markers reported by Lee et al. (2015) in an independent set of semen-derived DNA samples from 125 additional males using targeted massively parallel sequencing (MPS) technology. These data were used to develop a prediction model for age in semen. A third independent set of semen-derived DNA samples from 54 men tested the prediction model’s performance.

## RESULTS

### MethylationEPIC 850K BeadChip array analysis

Methylation data were collected from MethylationEPIC 850K BeadChip array analysis in a discovery set of 40 bisulfite-converted DNA samples extracted from semen of volunteers with a mean age of 36.0 years ± 7.4 (SD). Two DNA samples failed quality control of bisulfite conversion efficiency and were excluded from further analyses, leading to a mean age of the 38 discovery samples of 35.8 years ± 7.5 (SD) (Supplementary Figure 1). Correlation analysis of the EPIC microarray data indicated very strong demethylation in promoter regions in comparison to the whole set of CpG sites analyzed (median level 8.8% vs 76.7%, Supplementary Figure 2). Analysis of all 866,091 CpG sites showed that age-related demethylation occurs inside gene regions more frequently than expected and is characteristic of 14,916 (60.6%) significantly age-correlated DMSs (*P*-value < 0.05, Supplementary Table 1). It is worth noting that 17,367 (70.6%) significantly age-correlated DMSs (*P*-value < 0.05) were identified among sites with a high level of mean methylation. However, when strongly correlated DMSs (*P*-value < 0.00001) was considered, the hypermethylated sites were only slightly more frequent than hypomethylated sites (41% vs 59%) (Supplementary Table 2).

In the first step, Pearson’s r correlation analysis (*P*-value < 0.00001) and use of false discovery rate (FDR) ≤ 0.05 allowed the selection of 31 candidate CpG age markers for semen (Supplementary Table 3). Multivariable linear regression on power transformed DNAm data, supported by Bayesian Information Criterion was used to identify the best age correlated DMSs and allowed the identification of the optimal set of ten age predictors for semen. Among these ten selected markers, univariable linear regression on power transformed DNAm data revealed the highest age correlation (Pearson’s r = 0.77 and r *=* 0.76) and the highest statistical significance in *TUBB3* and *EXOC3*, (*P*-value = 1.12×10^-8^ and *P*-value = 3.64×10^-8^, respectively). In this set, only the *TBX4* gene showed negative correlation with age (*P*-value = 3.37×10^-7^, Pearson’s r = -0.72, Table 1).

**Table 1:** Age correlation results of the univariable linear regression analysis for the ten best age correlated CpG markers selected from Infinium Methylation EPIC BeadChip array analysis of semen-derived bisulfite converted DNA samples from 38 men.

**Gene**	**Probe ID**	**Power transformation**	**Pearson's r**	**Pearson's r *P*-value**	**Pearson's Benjamini-Hochberg FDR**
*PPP2R2C*	cg02766173	-4.00	0.72	3.63×10^-7^	0.03
*EXOC3*	cg10528482	-4.00	0.76	3.64×10^-8^	0.01
*SH2B2*	cg00018181	-1.17	0.71	7.44×10^-7^	0.04
*IFITM2*	cg01886988	-4.00	0.71	5.67×10^-7^	0.04
*SYT7*	cg17147820	-1.49	0.73	1.60×10^-7^	0.02
*ARHGEF17*	cg09855959	-4.00	0.71	6.72×10^-7^	0.04
*TUBB3*	cg18701351	-4.00	0.77	1.12×10^-8^	0.01
*TBX4*	cg19862839	0.36	-0.72	3.37×10^-7^	0.03
*GALR2*	cg07909178	-0.20	0.71	7.46×10^-7^	0.04
*PALM*	cg17704154	-4.00	0.69	1.467×10^-6^	0.04

A preliminary prediction model using the ten best DMSs developed from Pearson’s r coefficient analyses after power transformation, showed high prediction accuracy with MAE of 1.2 years (RMSE = 1.5) and together these ten CpGs explained 94% of the age variation in the dataset (Supplementary Figure 3). This overestimated value was caused by the small number of samples used to train and test the model, and was verified by further validation testing and predictive modeling in independent samples.

Given that the ejaculates used for DNA extraction contain spermatozoa as well as somatic cells, such as epithelial cells, we checked for potential somatic cell interference in the discovery data set (N = 40) for which EPIC microarray analysis was performed, by assessing DNAm levels in *RTL1* (*DLK1* on the Infinium HumanMethylation450 BeadChip array) and *INS-IGF2* (*IGF2* on the HumanMethylation450 array). In contrast to white blood cells and epithelial cells, sperm cells show hypomethylation of *RTL1* and hypermethylation of *INS-IGF2* [31, 34, 35]. Analysis of 14 CpG sites in *RTL1* and 4 CpG sites in *INS-IGF2* revealed methylation levels typical of sperm cells in 77.5% of the samples (Supplementary Table 4). In the remaining samples, the DNAm data indicated a slight admixture of somatic cells. Therefore, we repeated all statistical analyses for selection of the optimal set of age predictors by only considering samples from which no signal of somatic cells was seen. This analysis revealed that 30 out of 31 previously identified markers remained significantly age-related with FDR ≤ 0.05. The marker cg19862839 (*TBX4*) lost statistical significance, indicating a potential significance for age prediction in the somatic fraction of ejaculates. Since our study aimed to develop a predictive model for age analysis in semen, which may contain small amounts of somatic cells, this marker was not removed from further modeling.

### Validation of the discovered age-correlated candidate DMSs

Validation of the ten selected CpG sites was divided into two stages. First, using pyrosequencing, we confirmed a statistically significant age correlation of the *GALR2*, *ARHGEF17*, *TUBB3*, and *PALM* genes identified by MethylationEPIC Microarray BeadChip data analysis, by analyzing semen samples from the independent validation dataset (N = 162 semen samples not used for marker discovery) (Supplementary Table 5). This allowed us to verify correctness of the microarray analyses. In the second part of the validation, the whole set of ten age-correlated DMSs selected at the discovery stage and 3 previously known semen age markers from Lee et al. (2015) [28] were analyzed via targeted MPS in semen-derived, bisulfite converted DNA samples of 125 independent male donors aged 26 to 56 years (mean age 40.5 ± 8.2 (SD)) which had not been used for marker discovery. This data collection method was chosen because targeted MPS is now the forensic technology with the highest multiplexing capacity, while using a different DNAm data collection method may affect predictive model accuracy, i.e., from method-to-method bias [36]. In addition, amplicon-based targeted MPS allowed us to extend the analysis of the 13 candidate CpG sites and additionally investigate adjacent CpG sites, allowing detection of 36 CpGs in total (Supplementary Table 6).

**Figure 1:**
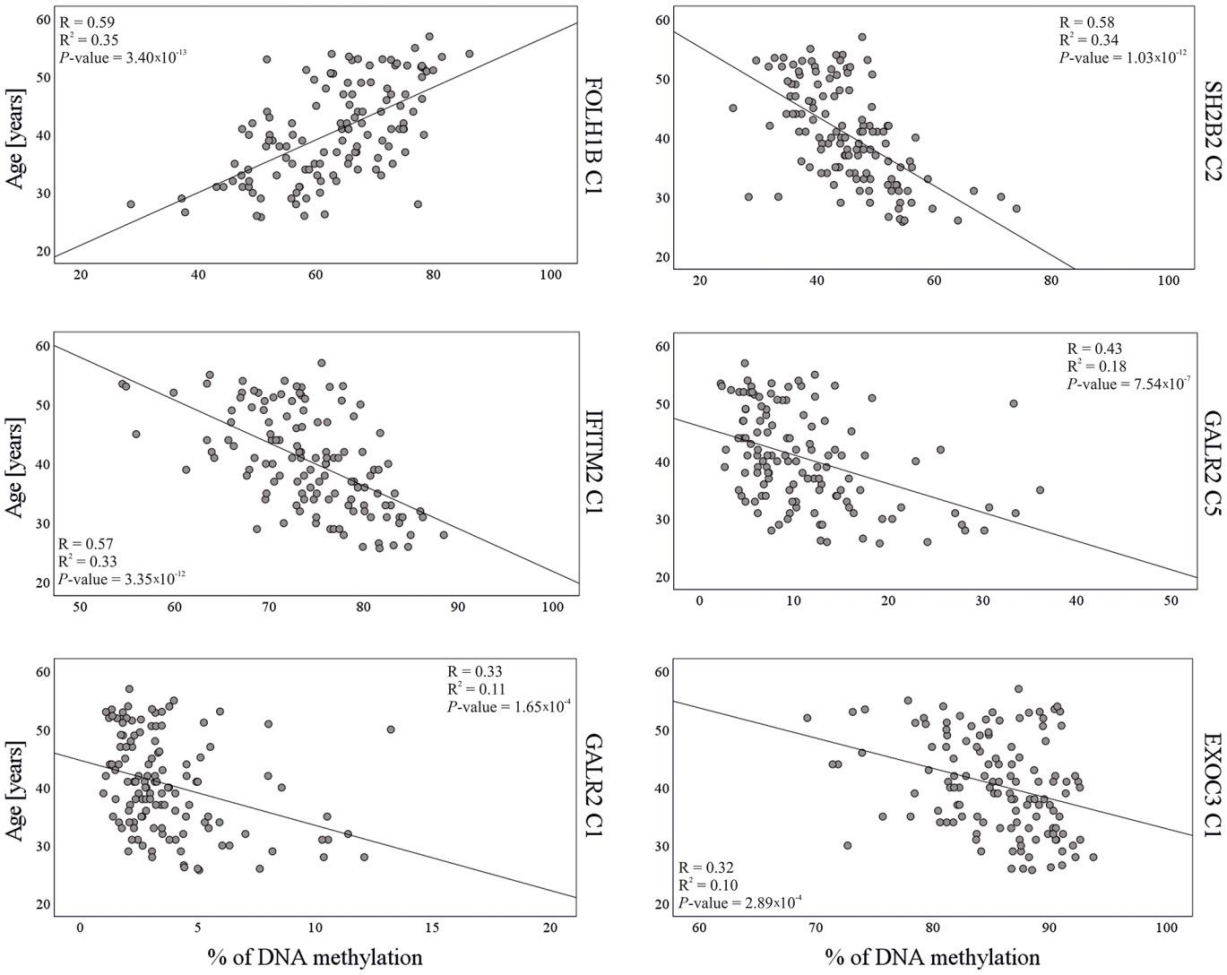
Correlation between DNA methylation and chronological age in the model training dataset (N = 125) for six CpG sites included in the final age prediction model for semen.

Univariable linear regression analysis revealed significant association (*P*-value < 0.05) with age, in 28 of the 36 (77.8%) analyzed CpG sites (Supplementary Table 6). Nine out of ten newly identified loci were successfully validated. The *TBX4* gene was the only locus not statistically significant in validation analysis. We also successfully replicated the age association of *FOLH1B*, *TTC7B* and *LOC401324* loci reported by Lee et al. (2015) [28]. Beta value analysis showed that, except for *FOLH1B* (standardized β value = 0.59), all statistically significant loci were negatively correlated with age (Supplementary Table 6). The highest statistical significance and strongest correlation with age (standardized β coefficient ≥ |0.5|) was detected in sites: *FOLH1B* C1 (β *=* 0.59, *P*-value = 3.40×10^-13^); *SH2B2* C2 (β *= -*0.58, *P*-value = 1.03×10^-12^); *IFITM2* C1 (β *= -*0.57, *P*-value = 3.35×10^-12^); *IFITM2* C2 (β *= -*0.57, *P*-value = 3.39×10^-12^); *SH2B2* C1 (β *= -*0.54, *P*-value = 1.00×10^-10^) and *GALR2* C8 (β *=* -0.5, *P*-value = 2.86×10^-9^) (Supplementary Table 6 and Figure 1). In univariable analyses, each of these DMSs explained 25–35% of the age variation observed in the analyzed semen samples (Supplementary Table 6). The highest statistical significance (*P*-value ≤ 5×10^-8^) was achieved for previously known genes *FOLH1B* (R^2^ = 0.35) and *TTC7B* (R^2^ = 0.24) plus newly identified genes *SH2B2* (R^2^ = 0.34), *IFITM2* (R^2^ = 0.33) and *GALR2* (R^2^ = 0.25). The *LOC401324* gene from Lee et al. (2015) was very close to this threshold (*P*-value = 5.08×10^-8^, R^2^ = 0.22, Supplementary Table 6).

### Age prediction modeling

**Figure 2:**
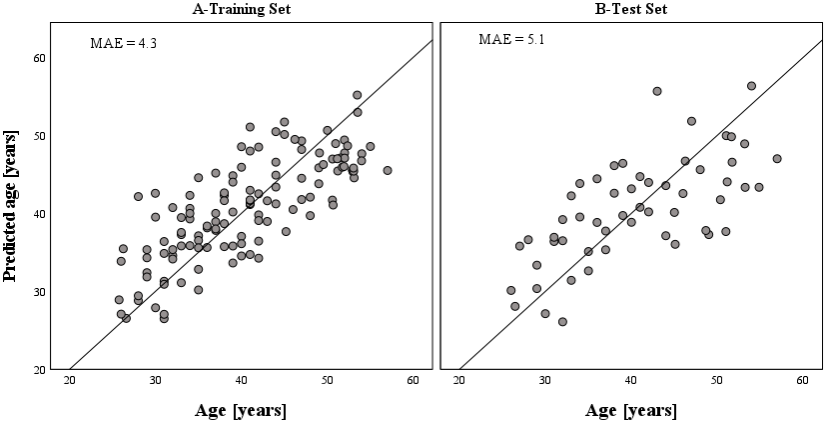
**Epigenetically predicted vs. chronological age in semen samples based on the model training (N = 125) and model test (N = 54) datasets, respectively.** The accuracy of prediction achieved with the developed epigenetic age prediction model for semen equals a MAE of 4.3 years (RMSE = 5.2) in the training set and a MAE of 5.1 years (RMSE = 6.3) in the test set. The six CpGs included in the model explained 60% of the age variation observed in the training set.

The dataset of 36 CpGs in thirteen independent loci obtained with 125 test samples in stage 2 validation testing, was further used for training purposes. Stepwise multivariable linear regression was used for variables selection and model building. Among the 36 CpGs, statistical analysis selected the six best age predictive DMSs from five genes (Table 2). The final age prediction model for semen included five DMSs from four novel genes *SH2B2*, *EXOC3*, *IFITM2* and *GALR2*, and one DMS from the previously identified *FOLH1B* gene [28]. These six markers together explained 60% of the age variation observed in the training dataset (Figure 2). The correlation with age of the six CpG sites included in the final model is shown in Figure 1. This model was then validated in a third independent model testing dataset of 54 semen-derived DNA samples collected from individuals aged 26–57 years (mean age 40.6 ± 8.5 (SD)) not previously used in the marker discovery, marker validation, or model building steps (Figure 2). The developed model predicted age in the training set with MAE of 4.3 years (RMSE = 5.2) and in the test set with MAE of 5.1 years (RMSE = 6.3) (Table 3 and Figure 2).

**Table 2:** Final set of age predictive CpGs in semen and characteristics of the multivariable linear regression model for semen.

**Gene**	**CpG no.**	**Probe ID**	**GRCh38**	**Standardized coefficient β**	**t**	**P-value**	**Adjusted R^2^**
*SH2B2*	C2	-	chr7:102288454	-0.43	-3.95	1.38×10^-4^	0.36
*FOLH1B*	C1	cg06979108	chr11:89589683	0.42	6.23	8.73×10^-9^	0.55
*EXOC3*	C1	-	chr5:525617	0.25	3.09	3.00×10^-3^	0.54
*IFITM2*	C1	cg05432003	chr11:312518	-0.30	-2.66	9.00×10^-3^	0.55
*GALR2*	C1	-	chr17:76077680	0.74	3.56	1.00×10^-3^	0.57
*GALR2*	C5	-	ch17:76077748	-0.61	-2.85	5.00×10^-3^	0.60

**Table 3:** Age prediction accuracy in semen using different epigenetic prediction models.

**Age prediction model**	**MAE (years)**			**RMSE (years)**	
	**Training set (N = 125)**	**Test set (N = 54)**		**Training set (N = 125)**	**Test set (N = 54)**
Current model (6 CpGs from 5 loci)	4.3	5.1		5.2	6.3
Lee et al. 2015 (3 CpGs from the *TTC7B*, FOLH1* and *LOC401324* genes)	4.9	5.7		5.8	7.0
Current model with novel markers only (5 CpGs from 4 loci)	4.3	5.2		5.5	6.3

*Present in the current model (6 CpGs from 5 loci).

In addition, we checked the accuracy of the model based on the three CpGs originally described by Lee et al. (2015) [28] in our test set and obtained MAE of 5.7 years. An alternative age prediction model based solely on our five new markers showed the same prediction error as the model covering all six DMSs (with MAE of 4.3 years) in the training set, but predicted age with slightly less accuracy in the test dataset (MAE of 5.2 years, Table 3).

## DISCUSSION

Standard STR profiling used in forensic DNA testing cannot resolve cases of sexual assault when the semen contributor’s STR profile is unknown (no suspects) to investigators, or the STR profile is not matched in the criminal DNA database. Mass DNA testing in these cases has previously proved to be useful but is difficult, especially when a large number of people are eligible for screening [37–40]. Directed by forensic DNA intelligence, such as epigenetic age prediction, mass DNA testing would be much more effective. Age is considered amongst the most useful items of information that can be used to narrow down the number of potential suspects. Several studies have demonstrated the potential of age prediction using DNA methylation analysis, but most have focused on predicting age in somatic cells, mainly blood [18, 41]. The available data indicate that developing epigenetic age methods for semen is more demanding. It has been shown that sperm DNAm levels increase with age across the genome [30], which is the opposite to observations in somatic cells [42]. Additionally, it has been reported that age markers discovered and validated in somatic tissues do not have predictive value in male germline cells [12].

### Discovery analysis

Use of the high-density MethylationEPIC BeadChip array in the present study enabled what is currently the most comprehensive screening of the entire epigenome, adding a large number of sites previously unavailable with HumanMethylation27 BeadChip and HumanMethylation450 BeadChip microarrays. This study led to the discovery of numerous age-correlated loci as candidate markers for epigenetic age prediction in semen. We have highlighted ten DMSs that showed the strongest correlation with age and had not been reported in previous studies [28, 29]. It is noteworthy that cg09855959 (*ARHGEF17*), cg18701351 (*TUBB3*), and cg17704154 (*PALM*) are not detected by HumanMethylation27 or HumanMethylation450 BeadChip microarrays and none of the ten markers selected by EPIC analysis are present on the 27K Infinium array. Lee et al. (2015) reported three age-correlated DMSs, while Jenkins et al. (2018) used DNAm data from their earlier studies [31, 43, 44] to select fifty-one age correlated regions in semen [29]. Our marker validation study involved 36 DMSs from thirteen regions in an independent dataset that was then used for model training. In addition to ten novel markers, we included three DMSs previously reported by Lee et al. (2015) [28]. The primary method of validation was MPS-based bisulfite amplicon sequencing. Using targeted MPS instead of using microarray data for marker validation and prediction modeling circumvents method-to-method bias associated with DNAm analysis - provided MPS is applied to future sample-of-interest analysis, such as forensic crime scene stains.

### Prediction modeling

The DNAm data obtained were used as the basis for developing an age predictive model for semen with six DMSs from *SH2B2*, *FOLH1B*, *EXOC3*, *IFITM2*, and *GALR2,* which predicted age with MAE of 5.1 years (RMSE = 6.3) in the model test dataset. The developed model is based on men aged 26-56 years and the test set had a similar age range (26-57 years). In real case analyses, younger and older people may be present, but as the model allows extrapolation these predictions can still be made. The error obtained is similar to that reported by Lee et al. (2015) (MAE 4.7 years) for their 3-CpG model. However, we used our data to develop a model based on 3 CpGs from [28] and this model predicted age in our test set with MAE of 5.7 years (Table 3). The difference may be related to the inter-population differences in DNAm variability [45]. An alternative model based solely on the five newly discovered DMSs revealed MAE of 5.2 years, which is only 0.1 year less accurate than the original model that includes a CpG from *FOLH1B*. This finding suggests *FOLH1B* provides important age information but is not crucial for epigenetic age prediction when used in the same model as the five novel age markers. It should be emphasized that apart from *FOLH1B*, the genes *TTC7B* and *LOC401324* selected by Lee et al. (2015), achieved very good results in our marker validation testing, providing independent confirmation of these gene’s correlation with age in sperm cells [28]. The MAE of our model is more than twice as inaccurate as the model reported by Jenkins et al. (2018) with MAE of 2.04 years in the training dataset, and a MAE of 2.37 years in the test set (N=10). [29]. However, Jenkins et al. used a much larger number of CpGs at 51 regions, requiring analyses for DNA methylation estimation at a much higher level than is viable from forensic DNA using current methods. The primary aim of our study was to develop a minimal marker model of practical utility in routine forensic analyses. In addition, our test set contained more samples over a wider age range, which could impact the accuracy estimate.

### The discovered age markers for semen

*FOLH1B* is the only gene in our final model previously described as an age predictor in semen [28]. Its usefulness has been recently confirmed in a Chinese sample set [46] and our study confirmed the utility of *FOLH1B* in a European population. The chromosome 11 *FOLH1B* gene encodes folate hydrolase 1b, also known as prostate-specific membrane antigen-like protein. Studies suggest *FOLH1B* may play an important role in the development and progression of prostate cancer [47]. Interestingly, *FOLH1B* is expressed in kidney and liver, but not in any other normal tissue, including prostate [48]. This gene is the top marker on our list of predictors and alone explains 35% of the variation (Supplementary Table 6). The remaining five genes included in the final model have not been previously correlated with age. The chromosome 7 *SH2B2* (also called APS) is the strongest age-correlated locus among the novel markers and is identified as a c-Kit-binding protein. Expression of *SH2B2* is restricted mainly to skeletal muscle, adipose tissue, and heart, while it may play an important role in insulin signaling [49, 50]. Notably, *SH2B2* has shown a differentially methylated CpG site in testicular injury in rats [51]. Univariable linear regression analysis revealed that *SH2B2* explains 34% of the variation (Supplementary Table 6). The chromosome 11 *IFITM2* gene encodes Interferon-induced transmembrane protein 2 and is activated against multiple viruses. Its IFN-stimulated gene expression is part of the response to infection with influenza A virus, SARS coronavirus (SARS-CoV), Marburg virus (MARV), Ebola virus (EBOV), and human immunodeficiency virus type 1 (HIV-1) [52, 53]. The chromosome 5 exocyst complex component 3 *EXOC3* gene is essential for the biogenesis of epithelial cell surface polarity [54–56]. This gene encodes a protein that is a component of the exocyst complex responsible for targeting vesicles to specific docking sites on the plasma membrane. The remaining two DMSs in the model are from the Galanin receptor type 2 (*GALR2*) gene and have R^2^ values of 0.18 and 0.11. The chromosome 17 *GALR2* gene encodes a protein involved in binding the hormone galanin and GALP, which results in signal transduction across the cell membrane in cooperation with G proteins [57, 58]. Its expression is mainly linked with the gastrointestinal tract, but is also detected in the testes [59].

### Possibility of epigenetic age prediction in semen

The moderate levels of age correlation and amount of age variation explained by the newly discovered DMSs confirms that epigenetic age prediction in semen is more complicated compared to age estimation in somatic cells. The individual effects of age predictors for semen as assessed by univariable linear regression analysis are smaller than those of somatic age markers in corresponding tissues. For instance, the highest R^2^ values for *FOLH1B* (R^2^ = 0.35) and *SH2B2* (R^2^ = 0.34) in our study are significantly lower than the strongest DNAm age predictor for blood *ELOVL2*. This predictor has shown consistently high R^2^ values ranging from 0.66 to 0.86 and correlation with age ranging from 0.85 to 0.92 in blood DNAm data [24, 60]. Notably, our results are consistent with those of Lee et al. (2015) for *FOLH1B*, who reported R^2^ = 0.44 for this gene. However, results for *TTC7B* and *LOC401324* in our data were weaker than those reported by Lee et al. (R^2^ = 0.24 vs. 0.61 and 0.22 vs. 0.60, respectively). It is important to highlight that Lee used East Asian samples and we studied Europeans. In addition, different values were reported by Li et al. (2020) for *FOLH1B* (R^2^ = 0.74) and *LOC401324* (R^2^ = 0.46) in Chinese and thus future research could reveal such inconsistencies are due to inter-population differences in DNAm levels. Consequently, age predictive models for blood based on just 4 to 7 CpGs show high accuracy with R^2^ = 0.94–0.96 and MAE = 3.1–3.9 years [13, 26, 61, 62]. In the case of sperm cells, a similar R^2^ = 0.89 and MAE = 2.37 was achieved by Jenkins et al. (2018), based on a predictive model from numerous CpGs in 51 regions [29]. Our 6-CpG model predicted sperm age with R^2^ = 0.60 and MAE = 5.1 years (RMSE = 6.3). Further research should evaluate our system and other age-related differentially methylated CpGs as potential markers of epigenetic age prediction of semen in different populations and using larger study cohorts [28, 29, 43, 63]. One drawback of our research is the lack of data in the youngest age group (under 26), where involvement in sexual offences is common. However, as discussed earlier, the model allows for extrapolation and age predictions in groups of younger individuals. Additional research samples will provide further improvements in our system. The discovery of age predictors in semen with stronger effects than those identified in this or previous studies, and by others before [28, 29], seems unlikely based on the current limited data. Therefore, increasing the accuracy of an age prediction model for semen will only progress by using much larger numbers of CpGs independently correlated with age in semen. This will require future technological advances in DNAm analysis for simultaneous typing of larger numbers of CpGs from low quality and low quantity forensic DNA.

In conclusion, we identified novel age correlated CpGs in ten genes previously not known to contain age-related DMSs, nine of which we successfully validated in an independent sample set. Our best model for predicting age from semen used six DMSs from five genes, of which four (*SH2B2*, *EXOC3*, *IFITM2*, and *GALR2)* were newly identified and *FOLH1B* was previously known. These six DNAm markers together explained ~60% of the age variation in the validation dataset, and the 6-CpG prediction model had an MAE of 5.1 years in our test dataset. The novel markers and model introduced here will be useful when applied to forensic cases, where knowledge of a semen donor’s age is unavailable but can be predicted with a practical and sensitive system from the crime scene semen samples.

## MATERIALS AND METHODS

### Semen samples

A total of 288 semen samples from volunteers were divided into four sets. The discovery set (N = 40, age range 24–58 years) was used for marker searches with the MethylationEPIC BeadChip array, with selections evaluated with the validation set (N = 162 samples, age range 26–60 years). Model building was made using 125 samples in the training set (age range 26–56 years), subsequently tested on a test set of 54 samples (age range 26–57 years). The validation set shared 67 samples with the training set and 26 with the test set (Supplementary Figure 1). Seventy-five semen samples were collected from patients from two Polish fertility centers: Medical Center Macierzyństwo and PARENS Fertility Center. Patients with severe oligoasthenoteratozoospermia were excluded from the study because of possible effects of this condition on DNA methylation patterns. Samples were collected based on the consent of the Bioethics Committee of the Jagiellonian University in Kraków no. 122.6120.78.2017 and 1072.6120.132.2018. All participants were informed about the goal of the study and signed consent forms to use the material for research purposes. Each semen sample was frozen and stored at -20° C until DNA extraction. DNA was extracted from 150 μl of semen using Sherlock AX Kits (A&A Biotechnology, Gdansk, Poland) according to the manufacturer’s protocol. The quality and quantity of DNA isolates were measured using NanoDrop 8000 UV-Vis Spectrophotometer (Thermo Fisher Scientific, Waltham, Massachusetts, USA, herein TFS) and Qubit 4 Fluorometer (TFS). An additional set of 213 DNA isolates was provided by the Institute of Human Genetics at the Julius Maximilians University in Würzburg, Germany. These samples were collected based on the consent of the ethics committee at the medical faculty of the University of Würzburg no. 212/15.

### MethylationEPIC 850K BeadChip array analysis

Whole-genome methylation profiles were obtained for the discovery set (N = 40) from men aged from 24 to 58 years old. All DNA samples were subjected to a quality check using 0.7% agarose gel electrophoresis. Bisulfite conversion and further epigenome-wide methylation analysis of these DNA samples was carried out using Illumina’s Infinium MethylationEPIC BeadChip array by the specialized Human Genomics Facility of Erasmus MC University Medical Center Rotterdam, The Netherlands. The data have been deposited in NCBI's Gene Expression Omnibus and are accessible through GEO Series accession number GSE179181 (https://www.ncbi.nlm.nih.gov/geo/query/acc.cgi?acc=GSE179181).

### Validation of age prediction markers for semen

In the first stage, four of the top ten markers selected based on microarray experiments were analyzed using the PyroMark Q96 MD pyrosequencing system in the validation sample set (N = 162) at the Julius Maximilians University in Würzburg. The statistical significance and correlation between chronological age and methylation percentage were calculated using linear regression with PS IMAGO PRO 5.1 (IBM SPSS Statistics 25). This analysis confirmed their correlation with age, and thus, in the next step, DNA methylation data was collected for the entire set of ten CpG candidates and the three CpG markers described in [28], using the VISAGE Enhanced Tool for age estimation from semen based on bisulfite amplicon MPS as described in Heidegger et al. (2021). The whole set of 13 candidate markers was analyzed in the training and test sets from donors aged 26 to 57 years (average age 40.5 ± 8.3 (SD)) from Würzburg (N = 144) and Kraków (N = 35). Briefly, 200 ng or 500 ng DNA was bisulfite converted with the EZ DNA Methylation-Direct Kit (Zymo Research, Irvine, CA, USA, herein ZR) according to the manufacturer’s protocol (DNA was eluted with 10 μl or 25 μl elution buffer, respectively). Amplification of the 13 markers was performed in two multiplex PCR assays using 4 μl of bisulfite converted DNA eluate, followed by a clean-up step with 1.5X Agencourt AMPure XP beads (Beckman Coulter, Brea, California, USA, herein Roche). Library preparation was performed using the KAPA HyperPrep Kit with the KAPA Library Amplification Primer Mix and KAPA Unique-Dual Indexed (UDI) Adapters (all Roche, Basel, Switzerland, herein Roche), as described in Heidegger et al. (2021). Library quantification was performed using KAPA Library Quantification Kit (Roche) and the QuantStudio 12K Flex Real-Time PCR System (TFS). Additionally, the specificity of PCR reaction and library preparation was checked with the 2100 Bioanalyzer Instrument (Agilent Technologies, Santa Clara, CA, USA). Finally, libraries were sequenced with two extra 0% and 100% methylation controls, Human Methylated and Non-Methylated WGA DNA Set (ZR), that were processed simultaneously. For sequencing, libraries were divided into two batches, pooled and prepared according to the MiSeq System Denature and Dilute Libraries Guide, Protocol A. A PhiX Sequencing Control (Illumina, San Diego, CA, USA, herein Illumina) in a final concentration of 5% was added to 12 pM of library pool for compensation of unbalanced nucleotide composition caused by bisulfite conversion. Sequencing was performed using the MiSeq FGx platform (Illumina).

### Data and statistical analyses

Infinium MethylationEPIC Array BeadChip data were pre-processed with R Bioconductor packages: “minfi”, “IlluminaHumanMethylationEPICanno.ilm10b4.hg19” and “lluminaHumanMethylationEPICmanifest” [64]. In the next step, methylation data were normalized with the SWAN method [65]. The DNA methylation data generated with MethylationEPIC 850K BeadChip array were subjected to statistical analysis. The preliminary candidate marker set was selected based on Pearson’s r correlation after application of the power transformation. Next, multivariable stepwise linear regression on power transformed data, supported by Bayesian Information Criterion was conducted within the R environment to identify the best semen age marker candidates [66]. Bioinformatic analysis of MPS data included quality assessment using FastQC, mapping of the bisulfite-seq reads to a custom-targeted reference with bwa-meth, SAM files sorting, conversion of the SAM files to BAM, and BAM indexing using Samtools. Depth of coverage in target regions was assessed using GATK (Genome Analysis Toolkit) [67] and each CpG’s methylation level was called based on the number of reads designated with the use of bam-readcount (with a minimum mapping quality of 30) (https://github.com/genome/bam-readcount). For this purpose, the number of C reads was divided by the sum of C and T reads. Only CpG sites with the minimum number of 1000 reads were accepted for further analyses, including the prediction modeling that followed. The generated DNA methylation data were subjected to statistical analysis, including univariable association testing and construction of the age predictive models with multivariable stepwise linear regression in cross-validation schema using PS IMAGO PRO 5.1 (IBM SPSS Statistics 25). Bayesian Information Criterion was used for model selection.

## Supplementary Material

Supplementary Figures

Supplementary Tables
