# Supplementary material for: Epigenetic age prediction in semen – marker selection and model development
Source: Aging (Albany NY). 2021 Aug 10;13(15):19145–64. doi: 10.18632/aging.203399 (PMC8386575; doi:10.18632/aging.203399)
Supplement: Supplementary Figures [file aging-13-203399-s001.pdf]

SUPPLEMENTARY FIGURES

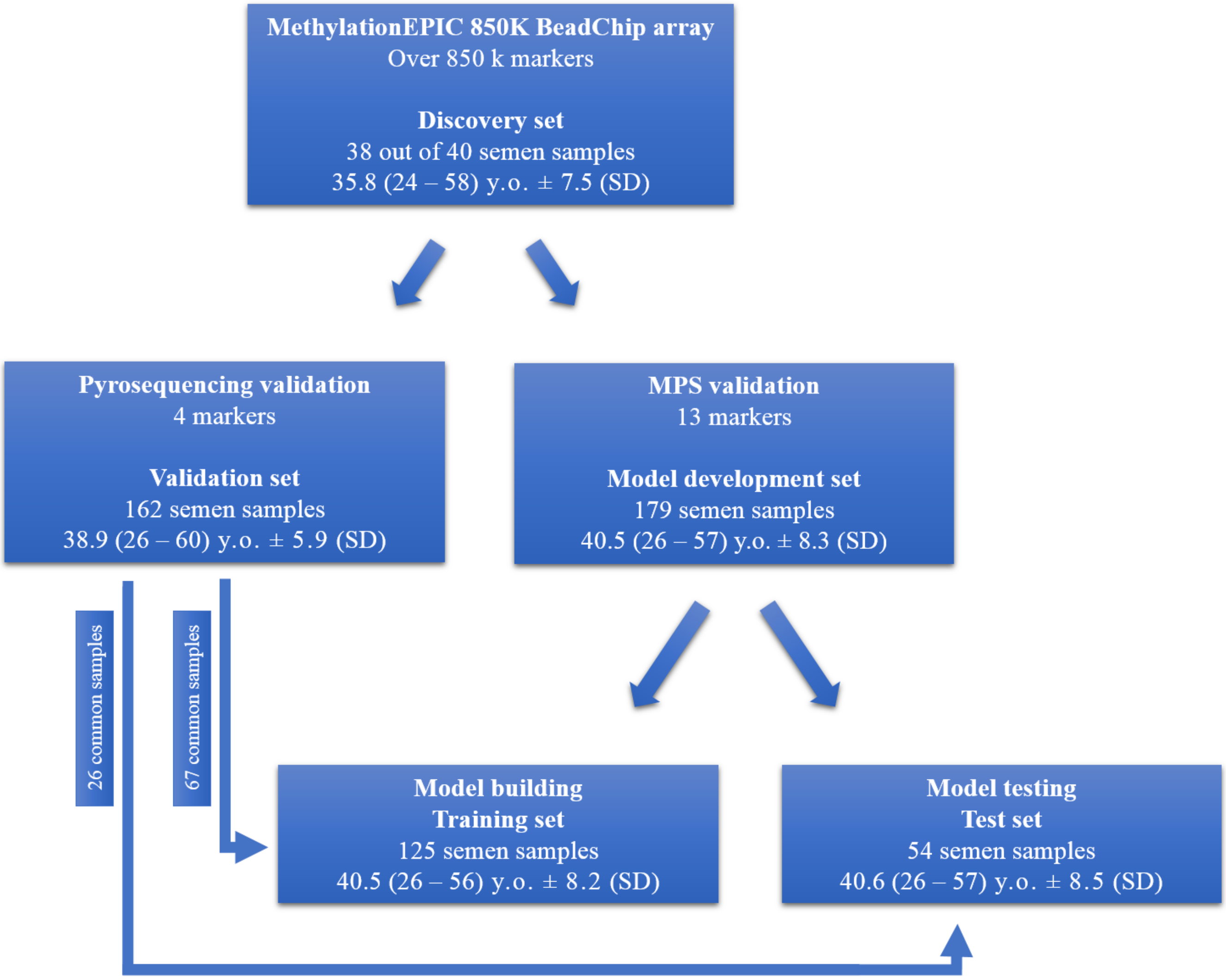

Supplementary Figure 1. Marker selection workflow and model development.

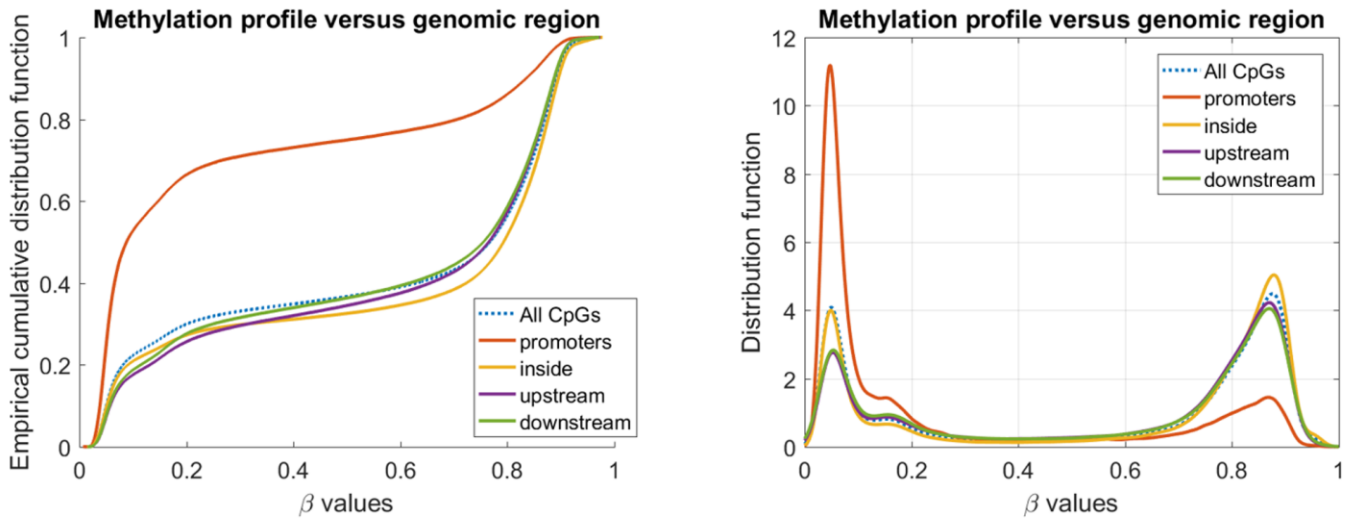

Supplementary Figure 2. Methylation profile in various genome regions.

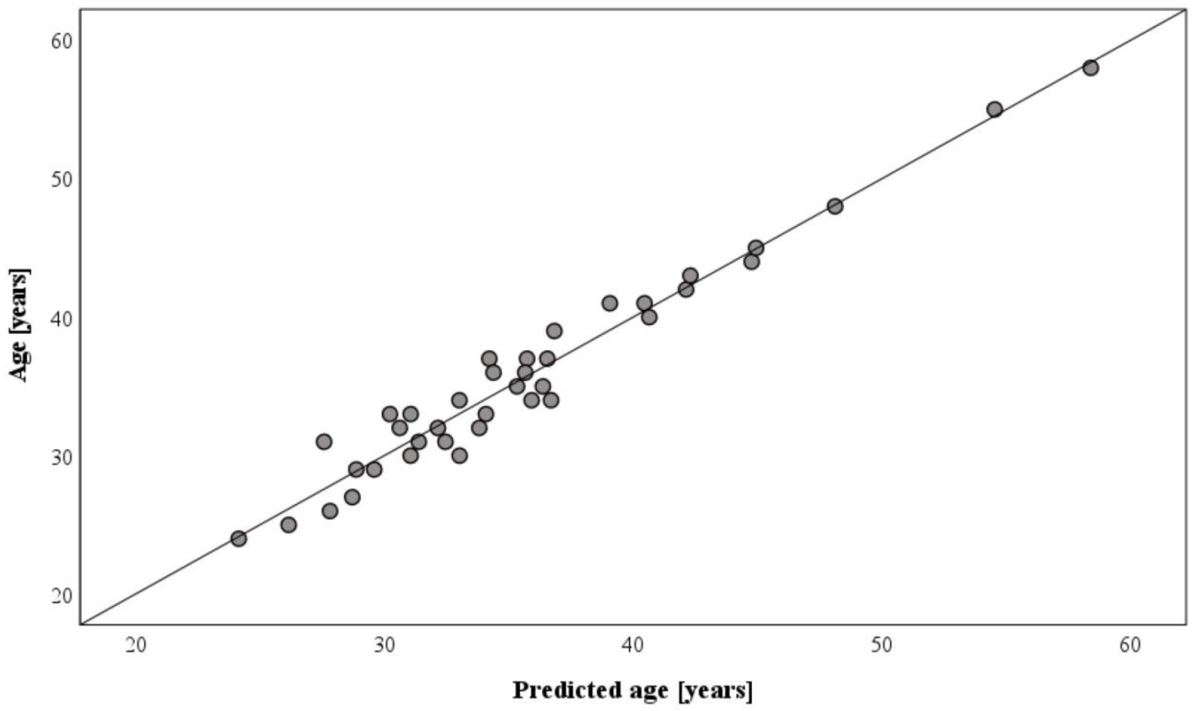

Supplementary Figure 3. Parameters of the initial predictive model based on 10 CpG markers. F-stat = 47.7; *P*- value = 3.56×10^{-14}; $R^2_{adjusted}$= 0.94; Absolute error for age prediction: Range: [0.06. 3.42]. Mean value: 1.20; Standard deviation: 0.93; RMSE = 1.5.
