# Supplementary material for: Epigenetic age prediction in semen – marker selection and model development
Source: Aging (Albany NY). 2021 Aug 10;13(15):19145–64. doi: 10.18632/aging.203399 (PMC8386575; doi:10.18632/aging.203399)
Supplement: Supplementary Tables [file aging-13-203399-s002.pdf]

SUPPLEMENTARY TABLES

Supplementary Table 1. Age-related DNA demethylation correlation analysis with regard to genomic regions.

Region	CpG sites		Significant association (*P*-value < 0.05)		Strong association (*P*-value < 0.00001)	
	[N]	[%]	[N]	[%]	[N]	[%]
Promoter	63.666	7.35	1.830	7.44	5	6.41
Inside	505.926	58.41	14.916	60.62	52	66.67
Upstream	140.149	16.18	3.417	13.89	7	8.97
Downstream	155.512	17.96	4.408	17.91	14	17.95
Close to 3’	838	0.10	36	0.14	0	0.0
Total	866.091	100	24.607	100	78	100

Supplementary Table 2. Correlation analysis of age-related CpG sites with regard to DNA methylation level.

Methylation level	CpG sites		Significant association (*P*-value < 0.05)		Strong association (*P*-value < 0.00001)	
	[N]	[%]	[N]	[%]	[N]	[%]
Low ($\beta \leq 0.5$)	317.618	36.67	7.240	29.42	32	41.03
High ($\beta > 0.5$)	548.473	63.33	17.367	70.58	46	58.97
Total	866.091	100	24.607	100	78	100

Supplementary Table 3. 31 CpG preliminary candidate markers identified based on Pearson's r and after power transformation calculated for 38 semen samples.

Probe ID	Gene	Region	Pearson's r
cg03650729	*TAL1*	inside	0.70
cg22820188	*LMNA*	inside	0.72
cg13502080	*ZAP70*	inside	0.69
cg02766173	*PPP2R2C*	inside	0.72
cg10528482	*EXOC3*	downstream	0.76
cg24603113	*C7orf50*	inside	0.69
cg00018181	*SH2B2*	inside	0.71
cg12108337	*FUT10*	downstream	0.71
cg07212803	*ARID3C*	promoter	0.70
cg08967938	*LHX3*	inside	0.70
cg13977355	*NRARP*	downstream	0.70
cg09899914	*NRARP*	downstream	0.75
cg11231500	*NRARP*	downstream	0.70
cg01886988	*IFITM2*	downstream	0.71
cg16543948	*AMBRA1*	downstream	0.72
cg17147820	*SYT7*	inside	0.73
cg09855959	*ARHGEF17*	inside	0.71
cg11703701	*HBQ1*	promoter	0.73
cg26939539	*SSTR5-AS1*	inside	0.70
cg23640964	*SSTR5-AS1*	inside	0.74
cg08958168	*SLC12A4*	inside	0.70
cg01420159	*OTX2-AS1*	inside	-0.72
cg18701351	*TUBB3*	inside	0.77
cg13006202	*TUBB3*	inside	0.76
cg18874912	*SMTNL2*	downstream	0.69
cg19862839	*TBX4*	inside	-0.72
cg07909178	*GALR2*	downstream	0.71
cg17704154	*PALM*	inside	0.69
cg12995604	*ZBTB7A*	inside	-0.71
cg01094301	*KLK6*	inside	0.70
cg06446412	*MIRLET7BHG*	inside	0.70

Supplementary Table 4. DNA methylation results from Infinium MethylationEPIC BeadChip array data (shown as average beta values) for 14 CpG sites from the *RTL1* gene and 4 CpG sites from the *INS-IGF2* gene calculated for the 40 tested samples.

Sample no.	*RTL1*	*INS-IGF2*	Sample no.	*RTL1*	*INS-IGF2*	Sample no.	*RTL1*	*INS-IGF2*	Sample no.	*RTL1*	*INS-IGF2*
1	0.20	0.90	11	0.12	0.89	21	0.12	0.89	31	0.13	0.89
2	0.13	0.90	12	0.12	0.88	22	0.15	0.89	32*	0.52	0.74
3	0.11	0.88	13*	0.64	0.73	23*	0.46	0.81	33*	0.59	0.75
4	0.15	0.90	14	0.14	0.91	24*	0.39	0.78	34	0.12	0.88
5	0.22	0.89	15	0.19	0.90	25	0.14	0.87	35*	0.35	0.80
6	0.14	0.87	16	0.29	0.87	26	0.13	0.89	36*	0.45	0.78
7	0.15	0.89	17	0.14	0.89	27	0.10	0.87	37*	0.31	0.84
8	0.18	0.89	18	0.10	0.89	28	0.18	0.89	38	0.12	0.85
9*	0.47	0.78	19	0.14	0.88	29	0.16	0.91	39	0.18	0.87
10	0.11	0.90	20	0.13	0.90	30	0.14	0.89	40	0.22	0.88

*Samples with a slight admixture of somatic cells.

Supplementary Table 5. Univariable correlation testing of a subset of CpG candidates using pyrosequencing.

Gene	GRCh38	Probe ID	Standardized Coefficient β	F stat	F stat *P*-value	R^2	No of samples
PALM	chr19:718608	cg17704154	-0.10	1.63	0.20	0.01	162
PALM	chr19:718625	-	-0.25	10.62	1.00×10^{-3}	0.06	162
GALR2	chr17:76077748	-	-0.29	15.01	1.56×10^{-4}	0.09	162
GALR2	chr17:76077752	cg19022866	-0.26	11.31	1.00×10^{-3}	0.07	162
GALR2	chr17:76077761	-	-0.23	9.08	3.00×10^{-3}	0.05	162
GALR2	chr17:76077795	cg07909178	-0.36	24.07	2.00×10^{-6}	0.13	162
ARHGEF17	chr11:73311506	cg09855959	-0.18	5.35	0.02	0.03	162
ARHGEF17	chr11:73311510	-	-0.16	3.95	0.05	0.02	161
ARHGEF17	chr11:73311527	-	-0.26	11.02	1.00×10^{-3}	0.07	161
TUBB3	chr16:89921897	cg18701351	-0.29	15.15	1.45×10^{-4}	0.09	162
TUBB3	chr16:89921901	cg13006202	-0.30	16.08	9.30×10^{-5}	0.09	162
TUBB3	chr16:89921921	-	-0.26	11.78	1.00×10^{-3}	0.07	162

Supplementary Table 6. Univariable correlation testing of CpG candidates using MPS technology.

Gene	CpG no.	GRCh38	Probe ID	Univariable association testing			
				Standardized Coefficient β	t	*P*-value	R^2
ARHGEF17	C1	chr11:73311483	-	-0.01	-0.11	0.92	0.00
ARHGEF17	C2	chr11:73311489	-	-0.02	-0.24	0.81	0.00
ARHGEF17	C3	chr11:73311506	cg09855959	-0.29	-3.39	9.38×10^{-4}	0.09
ARHGEF17	C4	chr11:73311510	-	0.07	0.80	0.43	0.01
ARHGEF17	C5	chr11:73311527	-	0.13	1.44	0.15	0.02
*EXOC3**	C1	chr5:525617	-	-0.32	-3.73	2.89×10^{-4}	0.10
EXOC3	C2	chr5:525656	cg10528482	-0.41	-4.94	2.48×10^{-6}	0.17
EXOC3	C3	chr5:525673	-	-0.44	-5.36	4.00×10^{-7}	0.19
EXOC3	C4	chr5:525680	-	0.04	0.49	0.63	0.00
*GALR2**	C1	chr17:76077680	-	-0.33	-3.89	1.65×10^{-4}	0.11
GALR2	C2	chr17:76077692	-	-0.29	-3.40	9.05×10^{-4}	0.09
GALR2	C3	chr17:76077717	cg08035416	-0.34	-4.07	8.44×10^{-5}	0.12
GALR2	C4	chr17:76077721	-	-0.40	-4.78	4.99×10^{-6}	0.16
*GALR2**	C5	chr17:76077748	-	-0.43	-5.22	7.54×10^{-7}	0.18
GALR2	C6	chr17:76077752	cg19022866	-0.41	-5.00	1.97×10^{-6}	0.17
GALR2	C7	chr17:76077761	-	-0.37	-4.40	2.30×10^{-5}	0.14
GALR2	C8	chr17:76077795	cg07909178	-0.50	-6.41	2.86×10^{-9}	0.25
*IFITM2**	C1	chr11:312518	cg05432003	-0.57	-7.73	3.35×10^{-12}	0.33
IFITM2	C2	chr11:312560	cg01886988	-0.57	-7.72	3.39×10^{-12}	0.33
LOC401324	C1	chr7:35260617	cg12837463	-0.46	-5.69	8.71×10^{-8}	0.21
LOC401324	C2	chr7:35260674	-	-0.46	-5.81	5.08×10^{-8}	0.22
*FOLH1B**	C1	chr11:89589683	cg06979108	0.59	8.16	3.40×10^{-13}	0.35
PALM	C1	chr19:718608	cg17704154	-0.20	-2.24	0.03	0.04
PALM	C2	chr19:718625	-	0.10	1.09	0.28	0.01
PPP2R2C	C1	chr4:6473419	-	-0.40	-4.71	6.93×10^{-6}	0.16
PPP2R2C	C2	chr4:6473429	cg07867360	-0.17	-1.97	0.05	0.03
PPP2R2C	C3	chr4:6473455	cg02766173	-0.36	-4.28	3.68×10^{-5}	0.13
SH2B2	C1	chr7:102288444	cg00018181	-0.54	-7.07	1.00×10^{-10}	0.29
*SH2B2**	C2	chr7:102288454	-	-0.58	-7.95	1.03×10^{-12}	0.34
SYT7	C1	chr11:61554783	cg17147820	-0.41	-4.97	2.22×10^{-6}	0.17
TBX4	C1	chr17:61466365	cg19862839	0.15	1.69	0.09	0.02
TTC7B	C1	chr14:90817262	cg06304190	-0.49	-6.22	7.22×10^{-9}	0.24
TUBB3	C1	chr16:89921880	-	-0.29	-3.36	1.00×10^{-3}	0.08
TUBB3	C2	chr16:89921897	cg18701351	-0.28	-3.21	2.00×10^{-3}	0.08
TUBB3	C3	chr16:89921901	cg13006202	-0.28	-3.22	2.00×10^{-3}	0.08
TUBB3	C4	chr16:89921921	-	-0.27	-3.05	3×10^{-3}	0.07

*Included in the final semen age model.
